# Liver Stiffness Value-Based Risk Estimation of Late Recurrence after Curative Resection of Hepatocellular Carcinoma: Development and Validation of a Predictive Model

**DOI:** 10.1371/journal.pone.0099167

**Published:** 2014-06-09

**Authors:** Kyu Sik Jung, Ji Hong Kim, Seung Up Kim, Kijun Song, Beom Kyung Kim, Jun Yong Park, Do Young Kim, Sang Hoon Ahn, Do Chang Moon, In Ji Song, Gi Hong Choi, Young Nyun Park, Kwang-Hyub Han

**Affiliations:** 1 Department of Internal Medicine, Yonsei University College of Medicine, Seoul, Korea; 2 Institute of Gastroenterology, Yonsei University College of Medicine, Seoul, Korea; 3 Department of Biostatistics, Yonsei University College of Medicine, Seoul, Korea; 4 Department of Surgery, Yonsei University College of Medicine, Seoul, Korea; 5 Department of Pathology, Yonsei University College of Medicine, Seoul, Korea; 6 Liver Cancer Special Clinic, Yonsei University College of Medicine, Seoul, Korea; 7 Liver Cirrhosis Clinical Research Center, Seoul, Korea; Queen Mary University of London, United Kingdom

## Abstract

**Background:**

Preoperative liver stiffness (LS) measurement using transient elastography (TE) is useful for predicting late recurrence after curative resection of hepatocellular carcinoma (HCC). We developed and validated a novel LS value-based predictive model for late recurrence of HCC.

**Methods:**

Patients who were due to undergo curative resection of HCC between August 2006 and January 2010 were prospectively enrolled and TE was performed prior to operations by study protocol. The predictive model of late recurrence was constructed based on a multiple logistic regression model. Discrimination and calibration were used to validate the model.

**Results:**

Among a total of 139 patients who were finally analyzed, late recurrence occurred in 44 patients, with a median follow-up of 24.5 months (range, 12.4–68.1). We developed a predictive model for late recurrence of HCC using LS value, activity grade II-III, presence of multiple tumors, and indocyanine green retention rate at 15 min (ICG R15), which showed fairly good discrimination capability with an area under the receiver operating characteristic curve (AUROC) of 0.724 (95% confidence intervals [CIs], 0.632–0.816). In the validation, using a bootstrap method to assess discrimination, the AUROC remained largely unchanged between iterations, with an average AUROC of 0.722 (95% CIs, 0.718–0.724). When we plotted a calibration chart for predicted and observed risk of late recurrence, the predicted risk of late recurrence correlated well with observed risk, with a correlation coefficient of 0.873 (*P*<0.001).

**Conclusion:**

A simple LS value-based predictive model could estimate the risk of late recurrence in patients who underwent curative resection of HCC.

## Introduction

Hepatic resection is the treatment of choice for hepatocellular carcinoma (HCC) in non-cirrhotic patients and remains the mainstay of treatment in cirrhotic patient with well-preserved liver function.[1] Although adequate patient selection, optimized surgical technique and postoperative management increased treatment efficacy, recurrence remains the main cause of a unsatisfactory long-term prognosis rate after resection of HCC.[2] Thus, identifying risk factors for recurrence after curative resection is important when selecting optimal candidates for resection and predicting a postoperative prognosis.

As recent study revealed that the prognosis and underlying mechanism are different for early and late recurrence, independent risk factors for each type of recurrence have been investigated respectively.[2]–[5] Among these, advanced liver fibrosis or cirrhosis is a known risk factor for late recurrence, as it contributes to multicentric recurrence at the remnant liver.[2], [3], [6] Furthermore, advanced liver fibrosis or cirrhosis is closely correlated with the development of postoperative mortality or complications, such as liver failure, worsening encephalopathy, or ascites.[7]–[9] Thus, a preoperative assessment of liver fibrosis severity is essential for prediction of postoperative outcomes.

Recently, liver stiffness (LS) measurement using transient elastography (TE, FibroScan) has been used to assess the degree of liver fibrosis noninvasively with high reproducibility and reliability.[10], [11] After much research into the role of LS value in predicting future development of clinical endpoints such as hepatic decompensation or HCC[12]–[14], several studies revealed that preoperative LS value could be used in the preoperative setting to predict postoperative outcomes such as hepatic insufficiency or operative blood loss.[7], [15] Furthermore, a recent study demonstrated that preoperative LS value was significantly correlated with intrahepatic recurrence after curative resection of HCC, particularly with late recurrence, suggesting that LS value could be used to stratify the risk of recurrence in patients with a remnant fibrotic liver.[16]

Thus, this prospective study was designed to investigate the correlation of LS value with the risk of late recurrence of HCC, and if such a correlation was identified, to construct and validate a LS value-based predictive model for late recurrence in patients who underwent curative resection of HCC.

## Materials and Methods

### Predictive model derivation

Between August 2006 and January 2010, patients with HCC, who were due to undergo curative resection of HCC at Severance Hospital, Yonsei University College of Medicine, Seoul, Korea, were prospectively enrolled in this study. Inclusion criteria were as follows; (1) age >20 years, (2) volunteered to participate in the study, (3) no previous diagnosis or treatment for HCC. TE was performed after enrollment to obtain preoperative LS value within 1 month prior to the operation by study protocol.

The database of our cohort included information on patient demographics, preoperative laboratory results, preoperative LS values, and pathological results of extracted liver and HCC specimens. The study protocol was consistent with the ethical guidelines of the 1975 Declaration of Helsinki. Written informed consent was obtained from each participant. This study was approved by the Ethics Committee/Independent Institutional Review Board of Severance Hospital, Yonsei University College of Medicine, Seoul, Korea.

### Diagnosis of HCC

The preoperative diagnosis of HCC was made based on dynamic imaging studies, biopsy, and alpha-fetoprotein (AFP) serology according to the AASLD guideline.[17] Before surgery, the size, number, and location of HCC, and the presence of extrahepatic metastatic lesions were confirmed by imaging studies including computed tomography (CT), magnetic resonance imaging (MRI), positron emission tomography, and angiography.

### Preoperative LS measurement

The principles of LS measurement have been described previously.[10] Briefly, TE generates an elastic wave using a vibrator ultrasound transducer applied to the intercostal spaces at the level of the right lobe of the liver and measures the propagation velocity of the shear wave, which is directly related to LS. LS values were obtained according to the instructions provided by the manufacturer. Immediately after ultrasonographic evaluation of the right hepatic lobe to avoid interference by HCC, LS measurement was performed on the right lobe of the liver through the intercostal spaces on patients lying in the dorsal decubitus position with the right arm in maximal abduction. A single experienced technician (more than 10,000 examinations), who was blind to the patients' clinical data, performed TE. The results were expressed as kilopascals (kPa).

Before surgery, TE was performed once at a single visit and LS values were measured more than 10 times during a single performance of TE. The interquartile range (IQR) was defined as an index of intrinsic variability of LS values corresponding to the interval of LS value containing 50% of the valid measurements between the 25th and 75th percentiles. The median value of successful measurements was selected as representative of the LS values in a given patient. Only LS values with at least 10 validated measurements, an IQR to median value ratio (IQR/M) of <0.3, and a success rate of at least 60% were considered reliable. LS value that did not satisfy these conditions was considered unreliable and excluded from further analysis.

### Surgery and follow-up

The time of surgery was determined after diagnostic work up of HCC considering the status of the patient and the operation schedule. All resections were performed by three experienced surgeons (GH Choi, KS Kim, and JS Choi). The type and extent of resection were determined according to tumor size, location, and liver reserve function estimated by the Child-Pugh score and indocyanine green retention rate at 15 min (ICG R15).[18] Intraoperative ultrasonography was performed routinely to determine tumor location and extent and to exclude the presence of additional lesions in the residual liver. Curative resection was defined as a negative histopathological surgical margin and the absence of residual tumor, as demonstrated by abdominal CT scans 1 month after hepatectomy.

Patients were followed up 1 month after surgery and every 3 months thereafter with tumor markers (AFP and des-gamma carboxy prothrombin) and imaging studies including abdominal CT or MRI. Recurrence was diagnosed based on the combined findings of these clinical examinations, and sub-classified as early (<1 year) and late (≥1 year) recurrence [3], [19].

### Histological evaluation of extracted liver specimens

A histological evaluation of the extracted liver specimens was performed by one experienced hepatopathologist (YN Park) who was blind to the patients' clinical information. Gross tumor classification, tumor size and number, tumor capsule formation and invasion, macroscopic or microscopic vascular invasion, satellite nodule, and the Edmondson-Steiner grade were determined.[20], [21] All HCCs were histologically confirmed in 139 patients and tumor stage was determined using the tumor, node, metastasis (TNM) staging system of the American Joint Commission on Cancer (7^th^ edition).[22] _ENREF_20_ENREF_20Liver fibrosis and necroinflammatory activity were evaluated semi-quantitatively in non-cancerous tissues according to the Metavir system.[23] Fibrosis was staged on a 0–4 scale: F0, no fibrosis; F1, portal fibrosis without septa; F2, portal fibrosis and a few septa; F3, numerous septa without cirrhosis and F4, cirrhosis. Histological activity grade (degree of necroinflammatory activity in the lobules and periportal area) was scored as follows: A0, no activity; A1, mild activity; A2, moderate activity and A3, severe activity. Activity grade was defined as lobule or periportal activity (whichever was higher) [23].

### Statistical analyses

Data are expressed as means ± standard deviation (SD), medians (range), or n (%), as appropriate. Comparisons between patients with late recurrence and those without were performed using Student's t-test or the Mann-Whitney test for continuous variables and the chi-square or Fisher's exact tests for categorical variables. In a univariate analysis, patient demographics including background liver etiology, preoperative laboratory results including ICG R15, preoperative LS values, surgical factors (types and extent of resections, resection margin, intraoperative blood loss, and perioperative transfusion) and pathological results of the extracted liver (fibrosis and necroinflammatory activity) and HCC (gross tumor classification, tumor size and number, tumor capsule formation and invasion, macroscopic or microscopic vascular invasion, satellite nodule, and the Edmondson-Steiner grade) were evaluated in terms of their relationship with late recurrence. Based on variables with clinical relevance and a univariate statistical significance, multivariate predictive model was constructed using a logistic regression model. The output of the model was expressed as regression coefficients, odd ratios (ORs), and 95% confidence intervals (CIs), which were used to predict late recurrence of HCC.

The model was validated by discrimination and calibration. Discrimination was assessed with the receiver operating characteristic curve, area under the receiver operating characteristic curve (AUROC), sensitivity, and specificity. We calculated the predicted risk of late recurrence based on our predictive model and then estimated the observed risk of late recurrence to assess calibration. Observed risk estimates were plotted against the predicted risk in the group to create a calibration chart. All statistical analyses were conducted using the SAS software, ver. 9.2 (SAS Institute, Cary, NC, USA).

## Results

### Baseline characteristics

**Figure 1:**
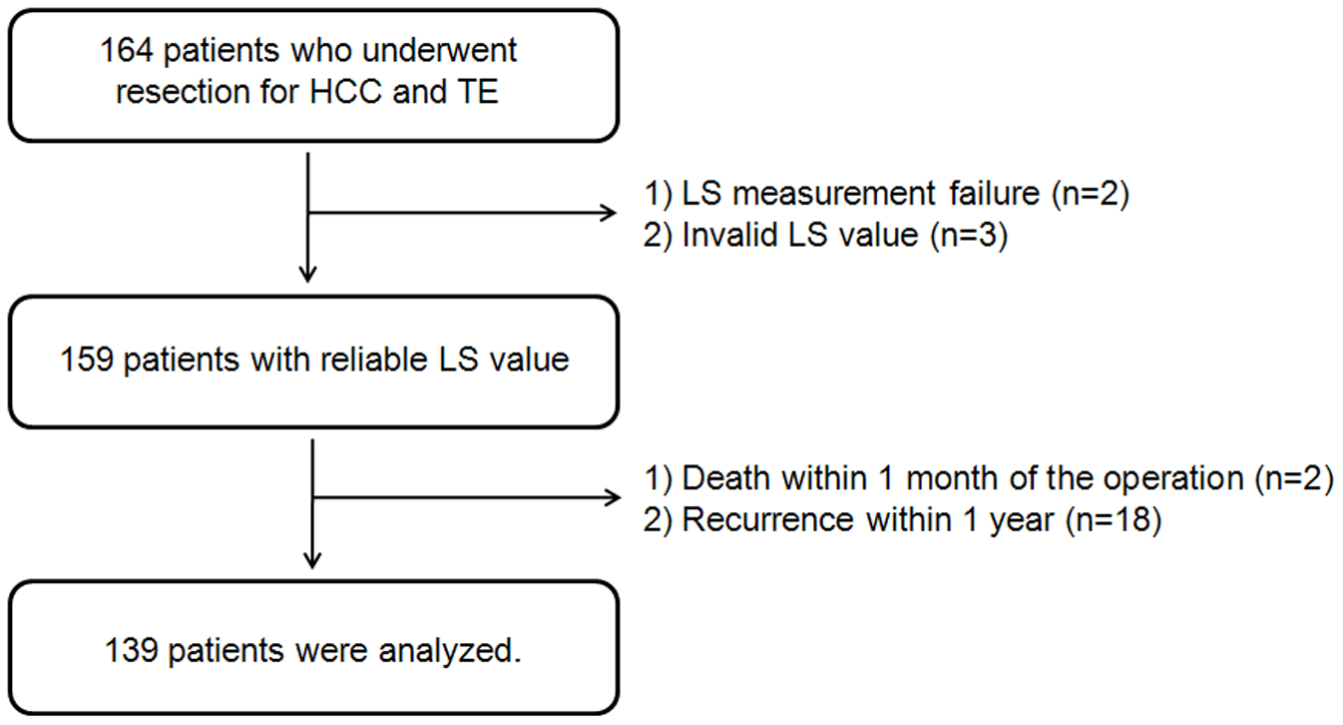
Flow chart of patient selection. A total of 164 patients with HCC undergoing curative resection were recruited. Five patients were excluded due to LS measurement failure or an unreliable LS measurement. Of the 159 patients with a reliable LS value, 20 were excluded due to postoperative death within 1 month, or early recurrence within 1 year. Thus, a total of 139 patients were selected for statistical analysis. HCC, hepatocellular carcinoma; LS, liver stiffness measurement.

A total of 164 patients with HCC who underwent curative resection of HCC were enrolled. Five patients were excluded due to LS measurement failure (no valid shot) or an unreliable LS value. Thereafter, since the purpose of our study was to investigate the risk factor and construct a model to predict late recurrence of HCC, two patients with perioperative mortality (death within 1 month of the operation) and 18 with early recurrence (<1 year) were also excluded from the analysis (**Fig. 1**). Finally, the study cohort consisted of 139 patients, who were used to develop the predictive model for late recurrence after curative resection. With exception of 16 patients who died during study period due to recurrence, the patients were followed-up until October 2012, and the median follow-up period was 42.9 months (range, 20.4–72.7 months).

The baseline characteristics of the study population are summarized in **Table 1**. Median age (119 males and 20 females) was 59 years. The most common etiology of underlying liver disease was chronic hepatitis B (n = 113, 81.3%). Liver cirrhosis was histologically confirmed in 56 (40.3%) patients. Median tumor size was 3.0 cm, and most tumors (n = 118, 84.9%) were single. According to the TNM staging system, 84 (60.5%) patients were classified as stage I, 7 (5.0%) stage II, and 48 (34.5%) stage IIIA.

**Table 1:** Baseline characteristics (n = 139).

Variable	
**Host factors**	
Age (years)	59 (32–80)
Male	119 (85.6)
Body mass index (kg/m^2^)	23.3 (15.9–32.5)
Etiology, HBV/HCV/non-B non-C	113 (81.3)/11 (7.9)/15 (10.8)
Total bilirubin (mg/dL)	0.7±0.2
Albumin (g/dL)	4.3±0.4
Prothrombin time (%)	93.1±7.6
Aspartate aminotransferase (IU/L)	35.5±17.9
Alanine aminotransferase (IU/L)	40.0±30.1
Alpha-fetoprotein (ng/mL)	24.4 (1–83,000)
Des-gamma carboxy prothrombin (mAU/mL)	56.0 (5–2,000)
Indocyanine green retention rate at 15 min (%)	8.5 (1.0–31.2)
**Tumor factors**	
Tumor size (cm)	3.0 (1.0–9.5)
Tumor number, single/multiple	118 (84.9)/21 (15.1)
Tumor stage, I/II/IIIA	84 (60.4)/7 (5.0)/48 (34.5)
Portal vein invasion	9 (6.5)
Satellite nodule	3 (2.2)
Edmonson-Steiner grade, I-II, III-IV	97 (69.8)/42 (30.2)
Non-tumor liver pathology	
F0-1/F2/F3/F4	5 (3.6)/44 (31.7)/34 (24.5)/56 (40.3)
A1/A2/A3	47 (33.8)/89 (64.0)/3 (2.2)
**Liver stiffness measurement**	
Liver stiffness value (kPa)	10.5 (4.0–45.0)
Interquartile range (kPa)	1.6 (0.3–9.8)
Success rate (%)	97.0 (63–100)

Variables are expressed as median (range) or n (%).

HBV, hepatitis B virus; HCV, hepatitis C virus; kPa, kilopascal.

Late recurrence was observed in 44 (31.7%) patients at a median of 24.5 months (range, 12.4–68.1 months). Intrahepatic recurrence was identified in all patients with late recurrence. Among these patients, simultaneous extrahepatic metastasis with intrahepatic recurrence were identified in seven patients at the time of recurrence (lung, n = 4; bone, n = 3).

### LS values and the performance to predict liver fibrosis

**Figure 2:**
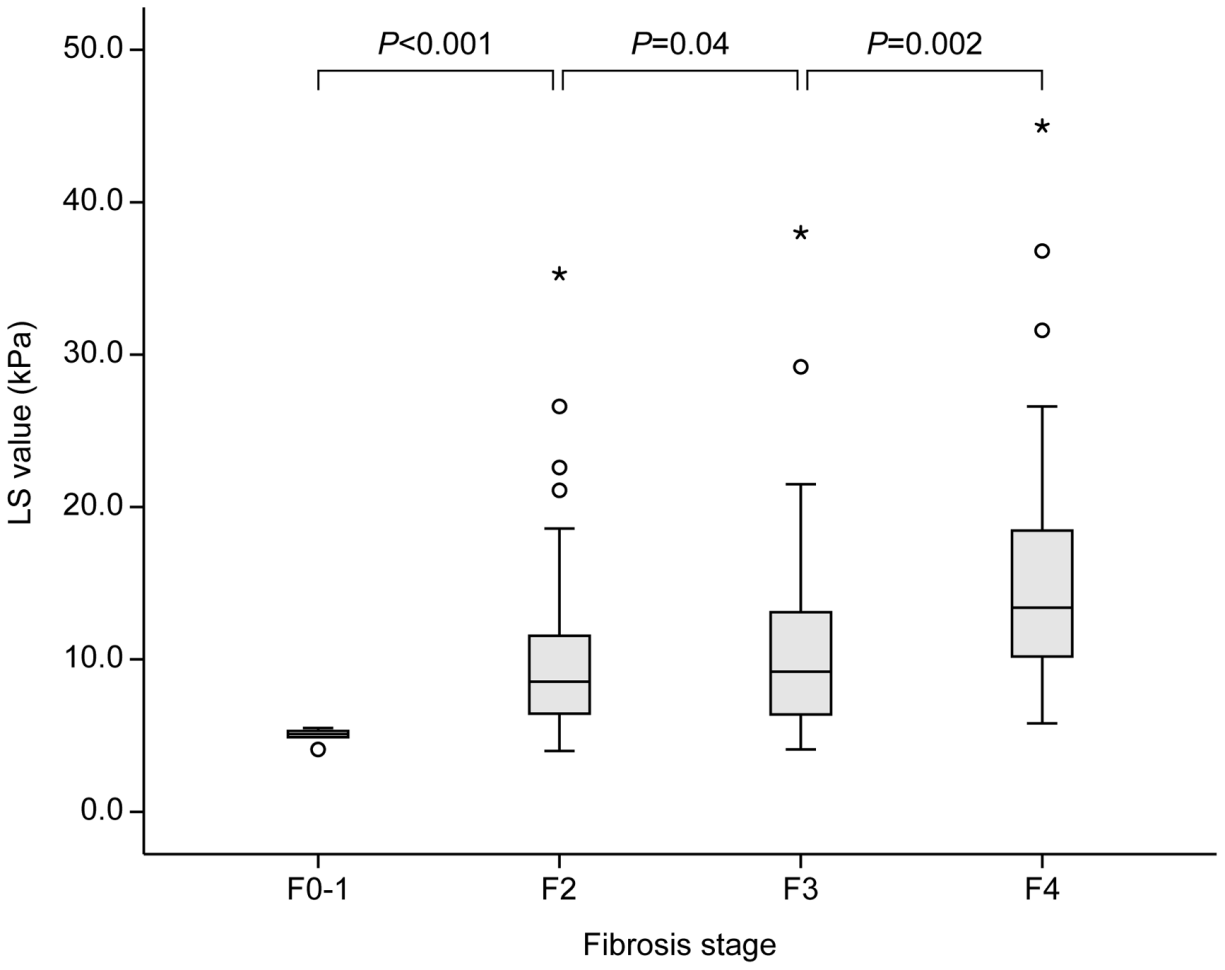
Distribution of LS values according to fibrosis stage. The median LS value increased significantly with fibrosis stage (P<0.001). LS, liver stiffness.

LS value using TE was obtained for all patients within 1 month before surgery (median 5 days; range 1–30 days). The median LS value was 10.5 kPa (range, 4.0–45.0 kPa). LS values according to fibrosis stage were 5.1 kPa for F0-1 (range, 4.1–5.5 kPa), 8.5 kPa for F2 (range, 4.0–35.3 kPa), 9.2 kPa for F3 (range, 4.1–38.0 kPa), and 13.4 kPa for F4 (range, 5.8–45.0 kPa) (**Fig. 2**). The median LS value increased significantly with fibrosis stage (all *P*<0.05). The AUROCs of LS values to predict ≥F2, ≥F3, and F4 were 0.951 (95% CI, 0.913–0.988), 0.796 (95% CI, 0.702–0.889), and 0.837 (95% CI, 0.755–0.919), respectively.

### Multivariate logistic regression model

The influence of each variable was tested in a univariate analysis (**Table S1**). In our cohort, ICG R15, the presence of multiple tumors, activity grade II-III, and LS value were significantly related with late recurrence in the univariate analysis (all P<0.05), whereas other variables were not statistically significant. A multivariate analysis was performed using the variables that exhibited statistical significance in the univariate analysis. **Table 2** shows the β-regression coefficient estimates in the multivariate logistic regression model. In the multivariate analysis, activity grade II-III, and LS value were identified as independent predictors of late recurrence (all *P*<0.05), whereas ICG R-15 and the presence of multiple tumors did not achieve statistical significance (*P*>0.05).

**Table 2:** Multivariate analysis to identify independent predictors ofHCC occurrence.

Factor	Beta	*P* value	Odd ratios (95% CIs)
ICG R15	0.0213	0.5715	1.022 (0.949–1.100)
Multiple tumors	0.3750	0.4565	1.455 (0.542–3.904)
Activity grade II-III	1.2470	0.0121	3.480 (1.313–9.221)
Liver stiffness value	0.0616	0.0172	1.063 (1.011–1.119)

HCC, hepatocellular carcinoma; CIs, confidence intervals; ICG R15, Indocyanine green retention rate at 15 min (%).

### Development of a predictive model of late HCC recurrence

In addition to the two independent variables (activity grade II-III, and LS value), ICG R-15 and the presence of multiple tumors were also incorporated as constituent variables to develop a predictive model for late HCC recurrence, which is a known significant risk factor for late recurrence.[24], [25] This predictive model showed a fairly good discrimination capability, with an AUROC of 0.724 (95% CIs, 0.632–0.816).
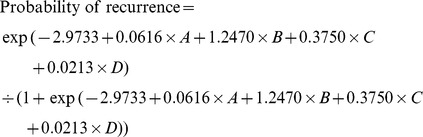
Where A = LS value (kPa); B = activity grade II-III (0: no, 1: yes); C = multiple tumors (0: no, 1: yes); D = ICGR15 (%)

### Discrimination and calibration

**Figure 3:**
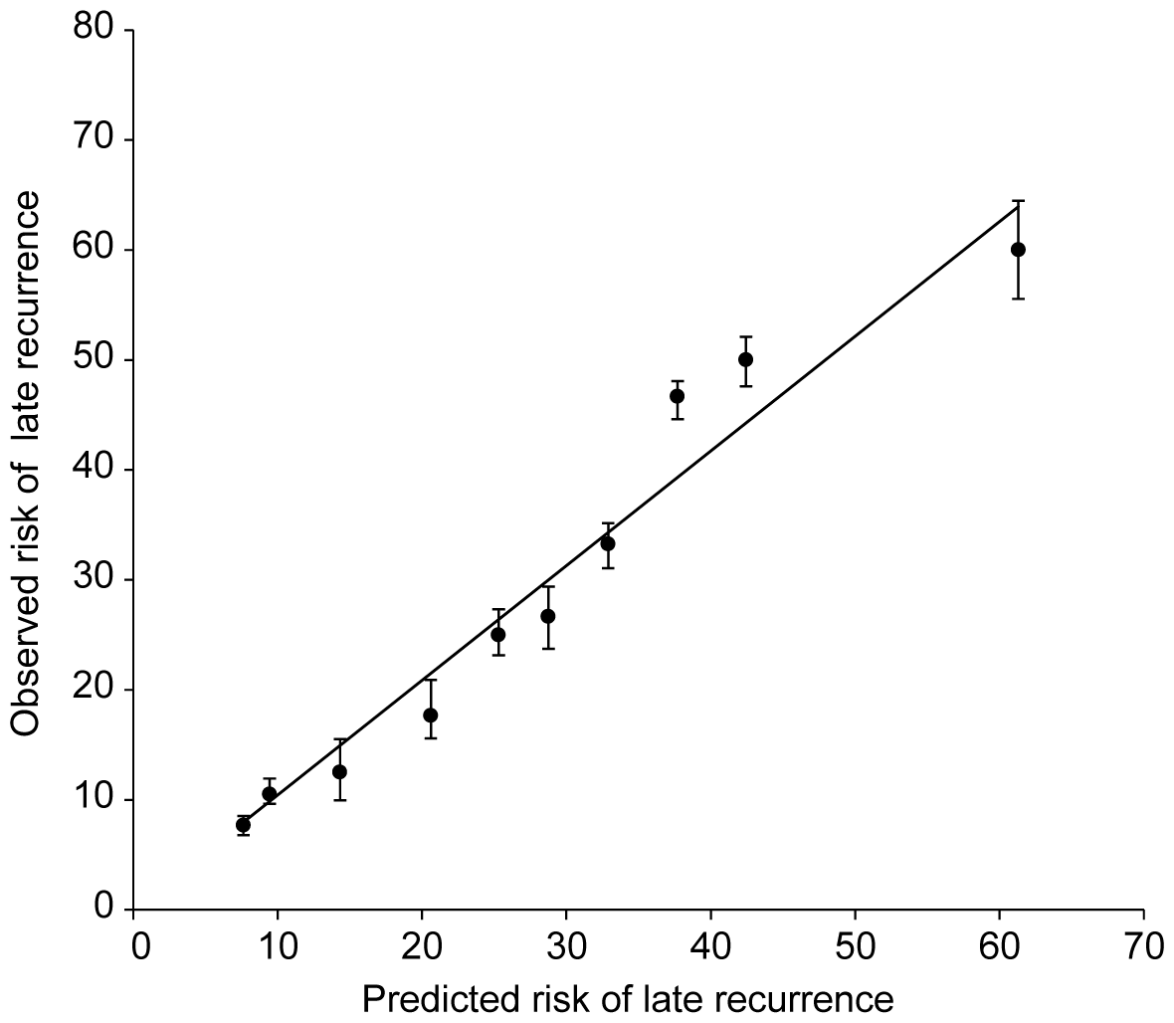
Calibration chart for predicted versus observed risk of late recurrence after curative resection of HCC. The predicted risk of recurrence calibrated well with the observed risk, with a correlation coefficient of 0.873 (*P*<0.001).HCC, hepatocellular carcinoma.

We used a bootstrap method to assess discrimination, in which 1,000 random samples were drawn with replacement from the original dataset, and AUROCs were recalculated in each sample. The AUROCs remained largely unchanged between iterations, with an average AUROC of 0.722 (95% CIs, 0.718–0.724). We plotted a calibration chart that compared the predicted and observed risks of late HCC recurrence (**Fig. 3**). The predicted risk of late recurrence calibrated well with the observed risk, with a correlation coefficient of 0.873(*P*<0.001).

## Discussion

Although the risk factors for postoperative recurrence have been investigated extensively, few mathematical models are available to predict recurrence after resection of HCC. [26]–[29] As none of the previous models included the effect of fibrosis in the formula, this is the first study to validate a specified prediction model for late recurrence of HCC based on LS value, which accurately reflects the burden of liver fibrosis. Finally, this study demonstrated that LS value-based predictive model could estimate the risk of late recurrence in patients who have undergone curative resection of HCC with considerable accuracy.

Compared to other risk factors, for a long time, the impact of liver fibrosis on recurrence has generally been underestimated. In previous studies, the influence of liver fibrosis was attenuated by other risk factors such as pathological factors, because recurrence was not classified into early and late one.[2], [28], [30] However, when types of recurrence were considered in the analysis, advanced liver fibrosis was selected as one of the independent risk factors for late recurrence.[3], [16], [24], [31] Additionally, as advances in surgical technique and proper patient selection have reduced early recurrence, which has a poorer prognosis than late recurrence, the effect of underlying fibrosis on long-term prognosis has gained greater importance. Despite its clinical importance, in most cases, it is difficult to assess histological staging of liver fibrosis prior to surgical resection. This suggests the need for a preoperative and non-invasive method of estimating liver fibrosis for selecting optimal candidates for resection or making individualized treatment and follow-up plans.

Recently, it has been proposed that preoperative LS value assessed using TE is significantly associated with recurrence, suggesting that clinicians can estimate fibrosis status noninvasively and accurately before surgery.[16] Consistently, preoperative LS value was selected as independent risk factor for late recurrence in this study, whereas histological fibrosis stage was not significant even in a univariate analysis.[16] These results might suggest that risk stratification using preoperative LS value could add more prognostic information for patients undergoing resection regardless of the histological findings, as LS might enable more precise quantification of liver fibrosis, expressed as continuous values, than simple stepwise histological grading. These findings also support a theoretical concept that the main mechanism of late recurrence is *de novo* development of HCC from a remnant fibrotic liver, rather than from dissemination of primary tumor cells [2].

Because the mechanism of late recurrence could not be explained solely by liver fibrosis, we developed a prediction model that included other important clinical factors and that could be validated internally. At first, as necroinflammatory activity was significantly associated with late recurrence in the multivariate analysis, consistent with previous studies, it was included in our predicting model.[24], [32], [33] Because liver fibrosis and necroinflammation are associated with a risk of carcinogenesis, it may enhance the performance to assess the risk of HCC development by incorporating simultaneously LS value and necroinflammatory activity into our prediction model.[4], [17] _ENREF_17Nevertheless, there can be a concern regarding potential overestimation of the influence of necroinflammation on LS values, which affected the overall performance of our prediction model.[34], [35] However, because we did not detect a correlation between LS and necroinflammatory activity in our patient cohort in a univariate analysis (*P*>0.05 by Spearman's correlation analysis), it was possible to use these two variables which reflect the degree of fibrosis and activity for our prediction model.

In addition, ICG R15, which had significant correlation with late recurrence in a univariate analysis, was included in our prediction model. Indeed, ICG R15 was significantly correlated with late recurrence in previous studies.[4], [24] _ENREF_24Although ICG R15 has been used to estimate remnant liver function after hepatectomy[18], the value of ICG R15 for predicting late recurrence was attenuated in our cohort. However, because ICG R15 could reflect the degree of functional liver status by estimating hepatic metabolic capacity whereas LS values could reflect the degree of physical status of liver fibrosis, we incorporated ICG R15 in our prediction model based on the hypothesis that the combination of these two parameters would strengthen the accuracy of our prediction model. Lastly, the presence of multiple tumors was included in our prediction models. Previous studies have demonstrated that the presence of multiple tumors was significantly related to late recurrence, suggesting that tumor multiplicity reflects increased carcinogenicity of the background liver.[2], [4] In this study, the presence of multiple tumors, which was confirmed histologically, was successfully diagnosed by radiological methods in all patients prior to resection. This result suggests that an advanced imaging modality can detect multiple tumors sensitively, thus the presence of multiple tumors, which was assessed by preoperative imaging, could be incorporated in the model.

Our new prediction model consisted of four variables: LS value, activity grade, presence of multiple tumors, and ICG R15, but other risk factors such as vascular invasion, tumor differentiation, and preoperative AFP level were not included in our model. Although these factors were identified as significant risk factors of recurrence in previous studies[36], [37], they did not achieve significance, even in the univariate analysis in this study. Additionally, because these factors did not enhance accuracy of our model, they were excluded from our final prediction model.

Although our study proposed a LS values-based HCC prediction model after curative resection, several issues remain unresolved in our study. First, this prediction model was developed using a relatively small sized cohort with various liver disease etiologies and validated internally and its accuracy seemed modest (AUROC = 0.724). Although, the etiology of liver disease was not significantly related to late recurrence in this study, consistently with the results of previous studies (P>0.05), further research with a larger and more homogenous study population should be followed by external validation.[2] Second, cross-sectional methods of identifying the risk factors for late recurrence in a cohort with variable follow-up time and exclusion of patients with early recurrence might lead to a selection bias. However, because the aim of our study was to develop a model to predict the risk of late recurrence itself, not the increasing risk of late recurrence over time, we used logistic regression instead of longitudinal statistical methods. Additionally, a short follow-up time less than 3 years was identified in 10 patients who died after recurrence and the remaining patients (n = 129) were followed up more than 3 years, suggesting that the current cross-sectional approach might be adequate for our cohort to compensate for the wide range of follow-up durations. Similar to our work, previous studies used cross-sectional methods to identify the risk factors for the late recurrence after resection of HCC.[3], [38] However, additional studies of a prediction model based on a survival analysis, such as Cox-regression or standardization of follow-up time, are required in the future. Third, as histological necroinflammatory activity grade was included, our model cannot be used prior to resection. Consequently, it does not seem feasible to use this model for stratification of an appropriate treatment modality such as liver transplantation, instead of surgical resection, especially for patients at high risk of recurrence after resection. Thus, it will be important to determine whether other noninvasive surrogate markers of the degree of necroinflammatory activity grade may be used as a preoperative risk calculator. Serum aminotransferase or apolipoprotein A1 are possible candidates as they revealed a significant correlation with necroinflammatory activity in a previous study.[39] Lastly, this model did not include gene expression profiles of surrounding non-cancerous liver tissue, which has been emphasized in recent studies as a significant risk factor for late recurrence after curative resection for HCC.[40], [41] However, from a different point of view, our results might suggest the importance of the characteristics of the remnant liver, such as LS values, ICG R15, and necroinflammation in predicting late recurrence in patients who have undergone curative resection of HCC.

In conclusion, this study constructed and validated a novel LS value-based model for predicting late HCC recurrence. This model, which involved tumor and non-tumor characteristics, demonstrated acceptable accuracy in patients who underwent curative resection of HCC. However, further studies with a larger sample size are warranted for external validation of our prediction model.

## Supporting Information

Table S1 **Comparison between patients with and without late recurrence.**
(DOCX)
