# Supplementary material for: Liver Stiffness Value-Based Risk Estimation of Late Recurrence after Curative Resection of Hepatocellular Carcinoma: Development and Validation of a Predictive Model
Source: PLoS One. 2014 Jun 9;9(6):e99167. doi: 10.1371/journal.pone.0099167 (PMC4049628; doi:10.1371/journal.pone.0099167)
Supplement: Table S1 — Comparison between patients with and without late recurrence. (DOCX) [file pone.0099167.s001.docx]

| **Table S1.** Comparison between patients with and without late recurrence | | | |
| --- | --- | --- | --- |
| Variables | Recurrence group | No recurrence group | *P*-value |
|  | (n=44, 31.7%) | (n=95, 68.3%) |  |
| **Host factors** |  |  |  |
| Age (years) | 60 (32-78) | 59 (34-80) | NS |
| Male | 39 (88.6) | 80 (84.2) | NS |
| Body mass index (kg/m^2^) | 23.3 (18.4-29.1) | 23.2 (15.9-32.5) | NS |
| Etiology, HBV/ HCV/ non-B non-C | 35 (79.5)/ 6 (13.6)/ 3 (6.8) | 78 (82.1)/ 5 (5.3)/ 12 (34.1) | NS |
| Total bilirubin (mg/dL) | 0.7 ± 0.2 | 0.7 ± 0.3 | NS |
| Albumin (g/dL) | 4.3 ± 0.4 | 4.3 ± 0.5 | NS |
| Prothrombin time (%) | 93.9 ± 6.8 | 92.6 ± 7.9 | NS |
| Aspartate aminotransferase (IU/L) | 40.3 ± 16.1 | 36.3 ± 18.4 | NS |
| Alanine aminotransferase (IU/L) | 44.1 ± 29.8 | 39.1 ± 29.9 | NS |
| Alpha-fetoprotein (ng/mL) | 26.8 (2.3-30,676) | 22.7 (1.0-83,000) | NS |
| Des-gamma carboxyprothrombin (mAU/mL) | 58.5 (5-2,000) | 56.0 (5-2,000) | NS |
| Indocyanine green retention rate at 15 min (%) | 11.8 (1.0-31.2) | 8.7 (2.8-24.8) | 0.0303 |
| **Tumor factor** |  |  |  |
| Tumor size (cm) | 3.0 (1.0-7.7) | 3.1 (1.2-9.5) | NS |
| Tumor number, single/ multiple | 32 (72.7)/ 12 (27.3) | 86 (90.5)/ 9 (9.5) | 0.0015 |
| Tumor stage, I/ II/ IIIA | 24 (54.5)/ 5 (11.4)/ 15 (34.1) | 60 (63.2)/ 2 (2.1)/ 33 (34.7) | NS |
| Tumor capsule, present | 35 (79.5) | 70 (73.7) | NS |
| Portal vein invasion | 4 (9.1) | 5 (5.3) | NS |
| Microscopic vascular invasion | 2 (4.5) | 3 (3.2) | NS |
| Satellite nodule | 2 (4.5) | 1 (1.1) | NS |
| Edmonson-Steiner grade, I-II/ III-IV | 15 (34.1) | 27 (28.4) | NS |
| **Surgical factor** |  |  |  |
| Type of surgical resection, major/ minor | 27 (61.4)/ 17 (38.6) | 45 (47.4)/ 50 (52.6) | NS |
| Resection margin >1cm | 38 (86.4) | 77 (81.1) | NS |
| Intraoperative blood loss, mL | 963.6 ± 920.5 | 853.6 ± 872.7 | NS |
| Perioperative transfusion | 13 (29.5) | 20 (21.1) | NS |
| **Non-tumor liver pathology** |  |  |  |
| Fibrosis stage |  |  |  |
| F0-2/ F3-F4 | 16 (36.4)/ 28 (63.3) | 33 (34.7)/ 62 (65.3) | NS |
| Activity grade |  |  |  |
| A1/ A2-A3 | 6 (13.6)/ 38 (86.4) | 41 (43.2)/ 54 (56.8) | 0.0011 |
| **Liver stiffness measurement** |  |  |  |
| Liver stiffness value (kPa) | 15.8 ± 9.1 | 10.8 ± 4.4 | 0.0001 |
| Variables are expressed as mean ± SD, median (range), or n (%), | | | |
| NS, not significant; HBV, hepatitis B virus; HCV, hepatitis C virus; kPa, kilopascal.. | | | |
